# Evaluating a whole-school approach to addressing gender-based violence in Scottish secondary schools (Equally Safe at School): a study protocol for a type I hybrid effectiveness-implementation trial

**DOI:** 10.1136/bmjopen-2024-096596

**Published:** 2025-02-16

**Authors:** Claire Hamilton, Ruth Lewis, Carolyn Blake, Anthony Purvis, Caroline Vaczy, Manuela Deidda, Niamh Kerr, Lisa Waiting, Kathryn Dawson, Malachi Willis, Emma McIntosh, Rod S Taylor, Laurence Moore, Kirstin R Mitchell

**Affiliations:** 1 MRC/CSO Social and Public Health Sciences Unit, University of Glasgow, Glasgow, UK; 2 Health Economics and Health Technology Assessment, University of Glasgow, Glasgow, UK; 3 Rape Crisis Scotland, Glasgow, UK; 4 Robertson Centre for Biostatistics, University of Glasgow, Glasgow, UK

**Keywords:** Adolescent, HEALTH ECONOMICS, PUBLIC HEALTH, Randomised Controlled Trial, Schools, Gender-Based Violence

## Abstract

**Introduction:**

Equally Safe at School (ESAS) is a whole-school intervention to reduce gender-based violence (GBV) in secondary school. ESAS comprises self-assessment, student-led action group, two-tier staff training, curriculum enhancement and policy review. Schools set up key activities in Year 1 and embed them in Year 2. GBV, including sexual harassment, is common in secondary schools and disproportionately affects young women and lesbian, gay, bisexual, transgender and queer youth.

**Methods and analysis:**

We will evaluate the effectiveness, cost-effectiveness, mechanisms of action and implementation of ESAS. We will recruit 36 schools across Scotland. The evaluation comprises three linked studies:

Study 1: Pragmatic cluster randomised trial with 1:1 school allocation to either immediate ESAS intervention start (intervention schools) or 12-month delayed intervention start (control schools). Our primary outcome of student experience of sexual harassment will be measured at 12 months post-randomisation. Analysis of primary and secondary outcomes (student and school level) will be conducted on an intention to treat (ITT) basis comparing schools according to their original allocation.

Study 2: Mixed-methods evaluation. Study 2A: Longitudinal follow-up will assess primary, secondary and intermediate outcomes at baseline, 12 months and 24 months of follow-up. Study 2B: Systems and realist-informed process evaluation will assess intervention and control school context, fidelity, dose and reach, acceptability and actor response, and how this varies by school and students. We will also assess implementation processes and mechanisms of action (beneficial or harmful), including if and how change is embedded over time, and if and how ESAS helps schools leverage other assets and resources.

Study 3: Economic evaluation to assess the within-trial and longer term cost-effectiveness of ESAS.

The methods include surveys in three out of six year groups (Years 2, 4 and 6) in all schools (baseline, 12 months and 24 months of follow-up); interviews with staff, students and other stakeholders; activity observations; brief surveys with key actors and analysis of trial documentation.

**Ethics and dissemination:**

Ethical approval by University of Glasgow MVLS Ethics Committee (200220268). Findings will be disseminated via multiple channels to researchers, GBV and education sector stakeholders, study participants and the public.

**Trial registration number:**

ISRCTN29792495

**STRENGTHS AND LIMITATIONS OF THIS STUDY**

- First school-based trial in the UK to evaluate a whole-school approach to tackling gender-based violence in school, including full economic evaluation.
- Novel type 1 hybrid effectiveness-implementation trial design, applied to a school-based intervention in an evolving implementation context.
- Novel integration of realist and systems perspectives in mixed-methods evaluation.
- Participants and trial team are not blinded to intervention/control allocation. However the statistician undertaking primary analysis will be blinded.

## Introduction

 Sexual harassment is defined as ‘unwanted attention of a sexual nature that has the effect of degrading, humiliating, or offending’.^1^ It is one of the most common forms of gender-based violence (GBV) defined as ‘any form of violence used to establish, enforce or perpetuate gender inequalities and keep in place gendered orders’.^2^ Studies across the globe suggest that sexual harassment is common in secondary schools, with prevalence varying by context and study methodology.^3^,^5^

Existing research demonstrates causal associations between any type of sexual harassment victimisation at school and adolescent well-being and later life health, as well as economic and social outcomes.^6 7^ School-based sexual harassment has a stronger adverse impact on health than bullying and these effects are more notable in girls.^8^ Sexual harassment in school is also serious because it normalises sexual violence.^9 10^ In Britain, 10% of women (1.4% of men) report sex against their will, most commonly in late adolescence.^11^ The European Institute for Gender Equality recently estimated the societal cost of GBV across the European Union at €366 billion/year,^12^ and a 2019 report estimated the cost of domestic abuse in 2017 to be approximately £66 billion in England and Wales.^13^ The costs include lost economic output, use of the health system and criminal justice system and physical and emotional impacts on victims.^12^ Prevention of GBV, therefore, has significant public health and wider societal benefits.

### Interventions to address GBV in schools

The evidence on the effectiveness of school-based interventions to reduce sexual harassment is fragmented. Much more is known about interventions to address related problems—such as dating and relationship violence (DRV) and bullying—than is known about interventions focusing on sexual harassment and GBV in general. Reviews of DRV interventions find a focus on curriculum sessions and individual behaviour, with few interventions tackling structural influences.^14^ Overall, interventions have demonstrated impacts on knowledge and attitudes but there is weak evidence for effects on behaviour.^14^ In one of the few effective interventions, *Shifting Boundaries*, the building-based component (eg, increasing teacher/security presence in unsafe areas) was key.^14^ Disappointing results from curriculum-based health interventions have led to interest in whole-school approaches which seek to change the school environment as well as addressing broader upstream social, physical and cultural contexts for health and well-being.^15^ Reviews have established effectiveness of whole-school interventions focused on bullying.^16^ In the UK, the INCLUSIVE trial had small but significant effects on bullying,^17^ but there have been few studies outside North America and little reliable evidence on programmes that have sustained effects, why and for whom.^16^ The evidence base for whole-school approaches to GBV is nascent and focuses on goals of gender equality in low- and middle-income country settings.^18^ Researchers and policymakers have called for robust evaluations of whole-school approaches to GBV.^18^

### Rationale, aims and research questions

Adolescence is characterised by identity formation and initiation of intimate relationships and is a common period for onset of GBV; schools are key sites in which norms are established (including those normalising sexual violence), and in which sexual harassment is enacted.^19^ Perpetration of sexually coercive behaviours in adolescence is a key risk factor for perpetrating sexual violence in later life.^20^ A universal primary approach that addresses systemic drivers of inequalities, transforms gender norms and includes young men (and male teachers) as part of the solution is recommended.^9^ A specific focus on sexual harassment is important; previous conflation of sexual harassment with bullying in education policy and research has held back attempts to address root causes of gender inequality and gendered power differences.^8^

School-based GBV primarily stems from inequality between boys/men and girls/women^21 22^ but may also implicate sexual orientation or gender identity.^4 21 23^ Patriarchal and unequal attitudes in society shape norms in schools that excuse and enable GBV.^9 24 25^ Patterning of sexual harassment shows a continuum in which exposure to ‘milder’ forms such as ‘sexual jokes, gestures or looks’ increases risk of exposure to more invasive behaviours like sexual coercion.^4^ There is an association between experiencing and perpetrating sexual harassment, with boys perpetrating more than girls.^4^ Gendered ‘teasing’ normalised as ‘banter’ pressures boys to conform to hegemonic norms of masculinity supporting violence.^9 26^ In terms of sexual harassment victimisation, most studies find higher victimisation rates among girls; and in both boys and girls there is a link between perpetration and victimisation.^27 28^ Studies also suggest that gender minority students are more likely to experience sexual harassment victimisation than girls or boys.^23 29^

Equally Safe at School (ESAS) is a whole-school approach to prevent GBV and promote gender equality in secondary schools. All individuals within the school environment are actors and beneficiaries. Wider beneficiaries include individuals who avoid exposure to GBV due to changes in attitudes and behaviours of students in ESAS, and wider society benefits from reduced GBV-related harms (and associated health and social care, legal/sentencing costs and loss to workforce productivity/educational attainment). Schools meet the costs of delivering ESAS in terms of dedicated staff time. Full information is at https://www.equallysafeatschool.org.uk/

ESAS is underpinned by Markham and Aveyard’s theory of health promoting schools^30^ which states that student health can be achieved through capacity for practical reasoning and capacity for affiliation with others, and thus can occur via changes to school organisation, staff–student relationships and curriculum. Initial theorised mechanisms of sustainable change from the pilot study are: that staff and students view ESAS (and the link between gender inequality and GBV) as coherent; that they are actively and collectively involved in efforts to change school culture; that they develop skills to recognise and address GBV; and that they are rewarded for positive behaviours (eg, via positive feedback or positive experiences of reporting sexual harassment). These hypothesised mechanisms are derived from the social development model,^31^ theories of social capital^32^ and the general theory of implementation.^19^

ESAS was developed via a 6-year collaboration between University of Glasgow and Rape Crisis Scotland (RCS). The intervention development phase involved student/staff group interviews and stakeholder consultation. The resulting theory of change and draft intervention design were refined via consultations with voluntary and statutory stakeholders. The research team undertook small-scale formative evaluation research alongside a three-school pre-trial pilot implemented by RCS (2018–2021).^4 33^

The evaluation is taking place in an evolving implementation context. Following the onset of the study, the Scottish Government and Convention of Scottish Local Authorities (COSLA)’s Equally Safe Strategy for preventing and eradicating violence against women and girls introduced an action for all secondary schools to engage with the ESAS programme, in particular to register an ESAS account and for key staff to take the eLearning course.

The study aims to evaluate the effectiveness, cost-effectiveness, implementation and mechanisms of change of the ESAS intervention. We will address nine research questions (RQ):

*RQ1*: What is the effect of ESAS on student experiences of sexual harassment (primary outcome) and a priori secondary and intermediate outcomes? (RQ1A—after 1 year? RQ1B—after 2 years?)

*RQ2*: What is the effect of ESAS on school prevention of, and response to, GBV? (RQ2A—after 1 year? RQ2B—after 2 years?)

*RQ3*: Does ESAS have a differential impact depending on student age, gender, socioeconomic status (SES), ethnicity, sexual orientation or by school-level academic attainment or area deprivation or by other factors identified as important? (RQ3A—after 1 year? RQ3B—after 2 years?)

*RQ4*: Is ESAS delivered with good fidelity, reach/dose, acceptability and actor response, and how does this vary between schools, and between students/staff within schools?

*RQ5*: What GBV-relevant activities and initiatives take place in delayed start (control) schools?

*RQ6*: To what extent does ESAS enable schools to leverage other assets and resources in short and medium terms?

*RQ7*: What does ESAS cost (and what activities does it displace) compared with the outcomes, and what might be the long-term societal cost-effectiveness of this intervention?

*RQ8*: After 2 years, what is the prevalence of sexual harassment and how well do schools prevent and respond to it?

*RQ9*: What do study findings overall suggest about intervention theory of change and potential for ESAS to be implemented and be effective/cost-effective elsewhere (particularly other parts of UK)?

## Methods and analysis

### Study design

The study is a type I hybrid effectiveness-implementation trial.^34^ This study type involves testing an intervention while also collecting information on its delivery in a real-world situation.^34^

**Figure 1:**
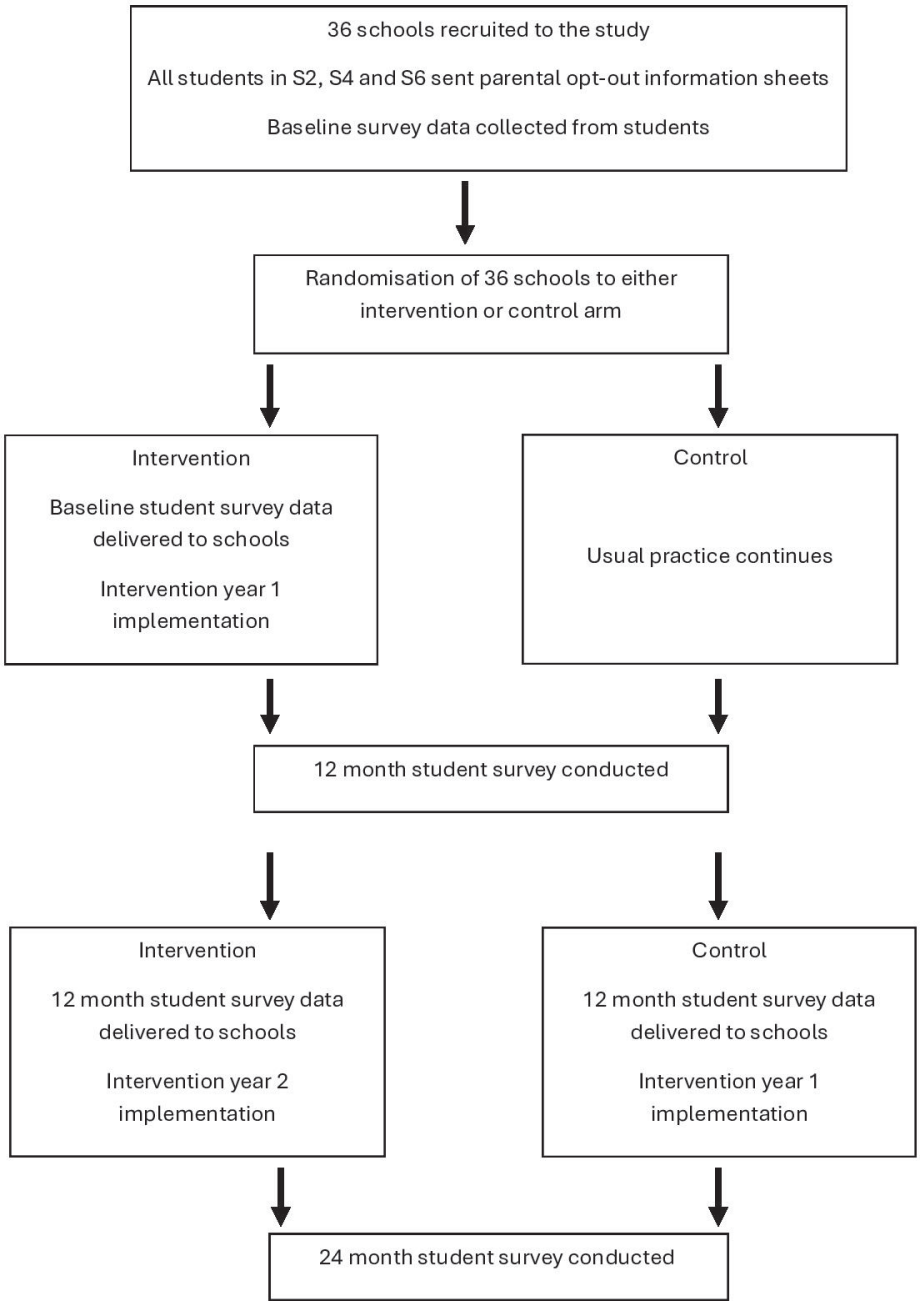
Flow chart showing intervention and control treatment.

The evaluation comprises three studies:

*Study 1. Pragmatic cluster randomised trial* in 36 schools in Scotland, randomised (1:1) to immediate start (intervention arm) or 12-month delayed start (control arm). Treatment as usual includes usual school initiatives on GBV and continues in both arms. Our primary outcome—student experience of sexual harassment—will be measured at 12 months. Analysis of primary, secondary and intermediate outcomes will be conducted on an intention to treat (ITT) basis comparing schools according to randomised allocation (see figure 1) (RQ1A; RQ2A).

*Study 2: Mixed-methods evaluation* based on realist and systems thinking. *Study 2A* (*longitudinal follow-up*) will assess primary, secondary and intermediate outcomes at baseline, 12 months and 24 months of follow-up (school level). *Study 2B* (*process evaluation*): Guided by realist and systems-informed frameworks for evaluating complex interventions,^35^,^39^ we will assess fidelity of intervention implementation, reach/dose, acceptability and actor response (RQ4); risks of contamination and any relevant ‘usual practice’ activities in control schools (RQ5); and implementation processes and mechanisms of action (beneficial or harmful), including if and how ESAS enables schools to leverage other assets and resources (RQ6), and if and how change is embedded over time (RQ9). We will collect some data from all schools and in-depth data in six case study schools. We will also collect data from wider education stakeholders and regionally based Rape Crisis (RC) staff involved in delivering ESAS training (RQ1B; RQ2B; RQ3–9).

*Study 3: Economic evaluation* will compare the intervention outcomes (consequences) with the costs of delivering the intervention and health and social care resource use, and, pending findings from Study 1 and Study 2, evaluate the likely long-term societal cost-effectiveness (RQ7).

### Setting

Secondary schools in Scotland provide comprehensive education to students aged 11–18 and comprise six academic years (S1–S6). All state-funded schools are mixed-gender. At the time of starting the study (July 2023), the ESAS intervention was available free of charge to all secondary schools in Scotland, and 12 had registered for an ESAS account and begun activities. Most schools in Scotland have policies and procedures in place to address bullying and harassment, but the extent to which these effectively address school culture and ethos with respect to GBV is highly variable or unknown. All schools are expected to work towards health and well-being indicators as part of the Scottish Curriculum for Excellence and many schools have undertaken self-assessment of the school environment under the ‘How Good is Our School’ quality framework. The Mentors in Violence Prevention (MVP) programme (https://education.gov.scot/improvement/practice-exemplars/mentors-for-violence-prevention-mvp-an-overview/) has been rolled out in most local authorities. The Scottish national leads of MVP and ESAS recognise their complementarity. While MVP focuses on peer mentoring, ESAS addresses wider school culture.

Scottish secondary schools meeting three criteria will be eligible to participate:

- 300 or more students on the school roll.
- Mixed-gender.
- Not previously undertaken an ESAS activity.

Schools with fewer than 300 students are excluded because they are atypical, and additionally because of the high unit cost of data collection. The trial will aim to recruit a sample that is varied in terms of region, rurality, size and area-level deprivation (measured by per cent of students receiving free school meals (FSM)). State schools will be recruited first; mixed-gender independent schools (up to four) will be offered the opportunity to take remaining places after December 2023 (end of first term of baseline data collection).

### Study population

For the cluster randomised trial, the study population is defined as students in Year 2 (S2, age 13), Year 4 (S4, age 15) and Year 6 (S6, age 17) at baseline and each follow-up. Each survey data collection point provides a ‘snapshot’ of the whole school and individual students eligible to participate differ at each administration according to their year group membership. The exception is students in Year 2 and Year 4 at baseline, who will be eligible to participate in the second follow-up survey when they will then be in Year 4 and Year 6 respectively. For Study 2B, the study population also includes all staff in participating schools, including ESAS lead staff and school leadership. Study 2B includes six case study schools in which all staff and students, and parents/carers are eligible to participate.

### Analytical sample and sample size for the pragmatic trial

We calculate that recruiting 36 schools (with a mean of 327 pupils/school and coefficient of variation of school size of 0.23, across three year groups assuming 75% response) provides 84% power to detect an 8% difference in the primary outcome of 35% (ie, relative risk: 0.77)^4^ in delayed start schools versus immediate start schools at 12 months of follow-up,^40^ assuming a moderate level of clustering (intracluster correlation coefficient (ICC) of 0.025, see online supplemental file 1) and an alpha of 0.05.

We remain adequately powered (ie, ≥80%) when we apply three potential scenarios to key sample parameters:

1. Attrition (loss of 10% schools (two per arm) at 12-month follow-up).
2. Lower baseline prevalence of primary outcome (32%).
3. Increased ICC of 0.05 to detect a primary outcome difference of 10.5% (ie, relative risk: 0.70).

Conservatively, our sample size calculation assumes no correlation in school primary prevalence between baseline and follow-up; a modest correlation (≥0.4) increases power to ≥90% in the base case (see online supplemental file 1) and would compensate for any reductions in power due to these reasons.

The estimated self-reported prevalence of primary outcome (endorsement of one or more sexual harassment experiences in the last 2 months) of 35% is derived from the pilot study (see primary outcome below). The calculation of ICC is based on the pilot study (online supplemental file 1).

### Recruitment and randomisation

We will recruit 36 schools across Scotland via a range of methods:

1. Email invitation to schools who have registered for an ESAS account but not yet logged any ESAS activities (ie, expressed a previous interest in ESAS).
2. Regular advertisements via the Scottish Schools Health Research Network (SHINE) and presentations at their annual conference.
3. Working with local authority education leads to advertise the opportunity to schools in their authority. Local authority education leads will be identified via the SHINE network, existing contacts of RCS and the research team, or via school leads.

Recruitment materials will include a brief for schools outlining the key study stages, including randomisation. School leads expressing interest in the evaluation study will meet with the principal investigator (PI) in person or via Microsoft Teams. They will be advised that participation in the evaluation involves randomisation to immediate or delayed start. Schools who are keen to do ESAS but do not wish to be randomised will be advised that they can implement ESAS outside of the evaluation and in their own timing. We will maintain a log of recruitment meetings and record reasons for non-participation. We will incentivise recruitment and retention by offering a £500 payment to all participating schools with an additional £1000 for case study schools, reflecting the greater research load on these schools. To promote retention, schools will be asked to sign an informal collaboration agreement outlining the roles and responsibilities of RCS, the research team and the school.

Formal recruitment is indicated by completion of the student baseline survey (October 2023 to December 2024). At this point, schools will be randomised (1:1) using minimisation to ensure balance between arms in:

1. Proportion of students receiving FSM.
2. School roll (size).
3. Whether they are currently undertaking MVP.

Schools will be randomised either to start the ESAS intervention immediately or to continue usual practice and start ESAS after 12 months (delayed start; control condition). The randomisation will be performed using centralised computer-generated randomisation sequence (Sealed Envelope) by trial statistician (AP).

All immediate start schools will be informed of the opportunity to be one of the six case study schools. Schools will be eligible to be case study schools if they have completed at least one of the five components of ESAS. Among schools willing to be case studies, we will purposively select for variation in size of school roll, %FSM/socioeconomic catchment area, rural/urban setting and MVP status.

### Intervention, comparator and possibility of contamination

ESAS is school led, with implementation guided by a web-based intervention hub with log-in and dashboard for registered schools. Schools use tools and guides on the dashboard and are supported by the national ESAS coordinator to undertake five activities (procedures):

(A) *Whole-school self-assessment* via online surveys and focus groups. This is done with management, staff and students to identify how gender inequality, adherence to stereotypes, and gendered bullying, harassment and abuse manifest in their schools. For schools taking part in the evaluation, data collected by the research team for baseline and follow-up surveys provide the student component of the self-assessment.

(B) *Student-ledaction group* supported by several supporting staff. The group should include students from a range of year groups and should be diverse, inclusive and reasonably representative of the wider student body. The group meets regularly, first reviewing the self-assessment findings and developing ideas to address the key issues. These form the group’s action plan.

(C) *Two-tier staff training*: School staff undertake training to develop capacity and capability to deal with issues relating to GBV. All school staff complete an eLearning module hosted on the ESAS hub. Staff in student support roles within the school additionally attend a 2-hour ‘enhanced’ in-person training session delivered by their local rape crisis centre.

(D) *Curriculum enhancement*: Staff can access a range of resources and lesson plans to support them to embed teaching about gender equality into the school curriculum to develop students’ understanding of issues relating to GBV and gender equality.

(E) *Policy review*: Schools review key policies and behavioural codes (such as promoting positive behaviour, equality and diversity, safeguarding) to ensure explicit, consistent and appropriate handling of issues relating to gender equality and GBV.

For the cluster randomised trial, the comparator is schools who wish to undertake ESAS but have been randomised to start the intervention 1 year later.

The ESAS intervention is funded under the Scottish Government’s national Equally Safe Strategy,^22^ is free to schools and is included in their whole school framework for preventing and responding to GBV.^41^ As such, there is potential for contamination if a control school started ESAS activities or other related whole-school initiatives. However, the delayed start schools are aware that they will shortly undertake the intervention and will usually have put ESAS in their school improvement plans for that year.

Schools in both arms continue with ‘usual activities’ which in this context may include the MVP intervention, discussions in personal and social development classes, sessions run by external providers and responding to reports of sexual harassment.

### Measures

Outcome measures (baseline, 12 months and 24 months).

#### Primary outcome

Our primary outcome measure is a binary measure of whether a student reports at least one episode of sexual harassment victimisation in the previous 2 months. This will be ascertained using a 5-item measure of sexual harassment victimisation in school settings that was adapted from the AAUW Sexual Harassment Survey (items 1–5)^42^ and DeGue *et al* (items 1–4) for their school-based cluster randomised controlled trial (RCT) of the ‘Effects of *Dating Matters* on Sexual Violence and Sexual Harassment Outcomes among Middle School Youth’.^40^ Refinements to wording were made during our within-trial pilot in one school. The five items are:

1. Receiving unwelcome sexual comments, jokes or gestures.
2. Being called gay, lesbian or trans in a negative way.
3. Being touched, grabbed or pinched in an unwelcome sexual way.
4. Being shown sexual pictures that they did not want to see.
5. Being blocked or cornered in a sexual way.

DeGue *et al* included another item from a separate measure to capture online sexual harassment (ie, being asked to do something sexual online when they did not want to).^40^ We will not include this item in our primary outcome in order to focus on behaviours that are more visible to other staff and students, and therefore more practical for schools to challenge, but we will retain this item in questionnaire as a potential measure of displacement effects. DeGue *et al*^40^ found that the internal consistency of their 6-item measure—when using a 4-month reporting timeframe—was between 0.64 and 0.74, and 47% of students reported at least one instance of sexual harassment victimisation. In our 2019 pilot (618 students across three schools) in which we asked about these five experiences of sexual harassment victimisation using a 3-month reporting timeframe, we found similar evidence regarding their reliability (Cronbach’s α=0.72) and that 44% of students reported at least one instance of sexual harassment victimisation in school during the previous 3 months. Evidencing the validity of this 5-item measure, we found that it was significantly associated in the expected directions with gender stereotypes, school attitudes and self-efficacy to make school safer.

Because we are interested specifically in sexual harassment that takes place in school or on the way to/from school, we will reduce the reporting timeframe to 2 months to maximise the chance that the reporting period will only cover term time (ie, if students were asked in October about school-based sexual harassment in the past 3 months, their responses would be biased given that part of the reporting period would have taken place during summer holidays). To account for this reduction in the reporting timeframe, we reduced our expected baseline prevalence of sexual harassment from 44% to 35% (which is reflected in our sample size calculation). In our within-trial pilot (98 students; one school), 37% of students reported at least one instance of sexual harassment victimisation in school or on the way to/from school during the previous 2 months.

#### Secondary outcomes

*Students’ experience of forced sexual activity* will be measured via single item: forced you to do something sexual (at school or the way to/from school in the last 2 months).^40^

*Students’ sexual harassment perpetration* will be measured using three items that assess engagement in unwelcome behaviours at or on the way to or from school in the last 2 months. The 4-month prevalence of perpetrating sexual harassment reported in De Gue *et al*^40^ was 29% (Cronbach’s α=0.73–0.83 across waves); the 3-month estimate from our pilot data was 27% (Cronbach’s α=0.62) (adapted from DeGue *et al*^40^).

*Students’ attitudes towards teen dating violence* will be measured using two items (adapted from Meiksin *et al*^43^).

*Students’ quality of life* will be measured using the Child Health Utility 9D (CHU-9D).^44^

*Students’ mental well-being* will be measured using the 7-item Warwick-Edinburgh Mental Wellbeing Scale (WEMWBS).^45^

*Students’ use of health and social services*. Questions on external health appointments and in-school health professional support (number of appointments of each service type in the last 3 months).

#### Intermediate outcomes

*Students’ perceptions of feeling safe at school* will be measured using two items that assess fear of sexual harassment and feeling unsafe at or on the way to/from school (adapted from Fisher *et al*^46^).

*Confidence to report problematic behaviours in school* will be measured using three items that assess students’ confidence in reporting three types of sexual harassment. These are original items for this study, tested in the pre-trial and within-trial pilot.

*Students’ endorsement of gender stereotypes* will be measured using three items (adapted from Meiksin *et al*, Rebecca *et al*, Flood and Kendrick^43 47 48^).

*Students’ endorsement of gender equal attitudes* will be measured using three items (adapted from Meiksin *et al*, Flood and Kendrick, Boxley *et al*^43 48 49^).

*Students’ self-efficacy to make the school safer* will be measured using three items that assess students’ perceived ability to help make their school a safer place. These are original items for this study, tested in the pre-trial and within-trial pilot.

*Students’ perceptions of staff and student response to sexual harassment at school* will be measured using five items; three of these items are based on a vignette describing a hypothetical incident of sexual harassment involving fictional students at their school. These are original items for this study, tested in the pre-trial and within-trial pilot.

*Students’ attitudes towards sexual harassment and GBV* will be measured using three items; two original items that were piloted and an item adapted from another study.^50^

*Students’ perceived ease of talking about GBV in school* will be measured using two items. These are original items for this study, tested in the pre-trial and within-trial pilot.

*Students’ perceived scope of sexual harassment as a problem at school* will be measured using three items that assess whether students think three types of sexual harassment are an everyday part of life in their school. These are original items for this study, tested in the pre-trial and within-trial pilot.

*Students’ perceptions of general school climate* will be measured using six items that assess students’ thoughts on belongingness, participation and commitment in their school. These are adapted from Sawyer *et al*^51^.

*Students’ perceived quality of teacher–student relationships* will be measured using five items, three of which are adapted from Sawyer *et al*.^51^ The other two are original items for this study, tested in the pre-trial and within-trial pilot.

#### Process measures

Process measures will be collected in all schools, with greater detail in immediate start schools and most detail coming from case study schools (see data collection methods).

*Context:* Context of schools in both arms of the trial, such as culture of student participation in decision-making, school-level student belonging, school leadership and organisational capacity, and other initiatives and priorities within schools.

*Fidelity:* Whether and how what is delivered in practice adheres to ESAS guidance for schools (*adherence*), including quality of delivery of the five intervention components (*quality*), and variations between and within schools, and if/how any local adaptations are either consistent with or undermine the programme theory.

*Dose and reach:* The quantity of intervention implemented in schools (*dose*), and extent to which members of the school community (management and staff, students, parents/carers) demonstrate awareness of, and engagement with, ESAS (*reach*), including variations within and between groups.

*Acceptability and actor response:* How systems actors within schools (students, senior leadership, school staff, parents/carers) respond to ESAS and perceive its acceptability; and how well it meets their needs, and variations between and within schools.

*Usual practice and contamination:* Any GBV-relevant school events, activities or policies that occur in delayed start schools before implementing ESAS. Any relevant contact between schools in different trial arms in Year 1 (eg, ‘good practice’ sharing between immediate start and delayed start schools).

*Mechanisms of action:* How intervention mechanisms interact dynamically with context (eg, school culture). Evaluative focus will be placed on assessing mechanisms hypothesised in the programme theory (eg, collective involvement by staff/students), alongside attention to identifying any unanticipated mechanisms. Informed by a complex systems perspective,^52^ we will examine whether and how ESAS becomes embedded within school systems, including conditions of *sustainable* change. We will explore how ESAS and school systems adapt and coevolve over time, including if/how ESAS enables schools to leverage other assets and resources (eg, schools’ participation in other GBV-relevant initiatives, such as MVP).

### Study data collection

#### Study 1/2A: assessment and follow-up

Student baseline and follow-up surveys will be administered to whole year groups (S2, S4, S6) by the study fieldwork team. Students will complete the web-based survey (programmed using Qualtrics^53^) on their mobile phones in class time, following a briefing video and after ticking a box to indicate their consent to participation. The fieldwork team will supply tablets and bring Wi-Fi routers as required. Ahead of the survey, students and their parents/carers will be sent a letter explaining the purpose and use of data and giving parents/carers the option to opt out their child. The surveys are anonymous but students in Year 2 and Year 4 will be invited, at baseline, to generate their own unique ID. This involves a set of instructions to students to create an ID using static and unique information that another person could not answer. The same set of instructions will be given at 24-month follow-up, allowing the surveys to be linked. This allows for nested cohort analysis (Study 2A), while maintaining real and perceived anonymity of questionnaire responses. We will ensure that younger students and those with additional support needs are given support and extra time as required; in our within-trial pilot the survey took 15–20 minutes to complete. Where possible, we will arrange a ‘mop up’ session using class lists to identify students absent at first administration. A summary report of the student survey is shared with school ESAS leads once they begin the intervention and serves as the self-assessment data for the school (outside of the evaluation study, schools collect this information themselves via the web-based dashboard).

#### Study 2B: process evaluation

We will maintain an evaluation monitoring log to capture recruitment and retention figures; notes from significant communications among the research team, the RCS ESAS national coordinator and schools; any adverse events; contemporaneous events, media coverage and other relevant information. Study 2B involves a range of qualitative and quantitative methods.

In *all* schools we will undertake:

- *Online survey of ESAS lead/s* (one survey per school) approximately 12 months after implementing ESAS (survey in immediate start schools ~12 months after baseline student survey; delayed start schools ~12 months after follow-up student survey).
- *Secondary analysis of data from school self-administered staff surveys* (Activity A in ESAS intervention) over 2 years in immediate start schools and 1 year in delayed start schools.
- *Analysis of school’s self-completed dashboard information* over 2 years in immediate start schools and 1 year in delayed start schools.

In six case study schools (all immediate start) we will undertake:

- *Small group discussions with school staff involved in implementing ESAS or handling incidents of reported GBV*; approximately two to three group discussions per school, ~4 staff per group (~48–60 staff total).
- *Small group discussions with students*; approximately four group discussions per school, ~4 students per group (~96 students total). One group to include action group members; other students recruited from spread of year groups.
- *Brief online survey for parents/carers.*
- *Structured observations of ESAS activities* (eg, enhanced staff training and action group meetings), two observations per school.
- *Workshops with staff and students to map the wider impacts of ESAS in school system over time*, ~2 workshops per school.
- *Review of school policies and any curriculum changes related to GBV* (Activities D and E in ESAS intervention).

We will also undertake:

- (*Where possible*) *interviews with ESAS lead staff in schools that do not engage with the intervention or that drop out.*
- *Interviews with RC staff (~4–6) based in local authorities* who are supporting the intervention (promoting it with schools and/or delivering enhanced training).
- (*Where possible*) *interviews with education stakeholders* (*~5–6*), including those based within local authorities.

#### Study 3: economic evaluation

The data for the economic evaluation will be collected via the student baseline and follow-up surveys. Measures include the CHU-9D instrument and a bespoke health economics questionnaire capturing a broad set of resource use (use of health and social services such as general practitioner (GP), school nurse, school counsellor). The cost of the ESAS intervention (actual cost; opportunity cost) and the comparator (ie, standard GBV activities part of school curriculum) will be collected via several sources, including school interviews, ESAS web-based dashboard, qualitative interviews and RCS.

### Analyses

#### Study 1: cluster randomised trial

*Primary analysis:* Analysis of primary and secondary outcomes at 12-month follow-up will be conducted on an ITT basis comparing schools according to randomised allocation, without imputation for missing data. The primary analysis for all outcomes will be a model adjusted for school-level baseline prevalence and minimisation variables (school size, percentage receiving FSM and whether schools are implementing MVP). Sensitivity analysis for all outcomes will compare cluster-level models and individual-level models with school included as a random effect. The cluster-level models may be more robust for dichotomous outcomes. Further subgroup and exploratory analysis may be conducted based on emergent data/findings from the process evaluation.

*Subgroup analysis:* Exploratory analyses will be undertaken to assess if ESAS has differential effects on 12-month primary and secondary outcomes by adding interaction terms for prespecified (1) school-level variables (including school size, percentage FSM, MVP status) and (2) student-level variables (eg, age/gender/SES). We will explore effects by measures of intervention dose/implementation.

Data analysis will be conducted and reported in accordance with Consolidated Standards of Reporting Trials extensions for cluster RCTs. ICCs for all outcomes will be reported by trial arm. Analyses will be performed by a statistician blinded to group allocation using STATA. A detailed statistical analysis plan (SAP) will be prepared prior to analysis and agreed with independent trial steering committee (TSC)/data monitoring and ethics committee (DMEC).

Key sources of missing data within the trial include: (1) school-level data due to a school withdrawing participation from the study, (2) missingness of specific pupil responses to questions in each survey round and (3) missingness of self-generated pupil IDs for the longitudinal follow-up at 24 months. Suitable approach methods for handling each of these sources of missingness will be developed and finalised prior to data analysis. Sensitivity analyses will be conducted to assess the potential effect of these sources of missing data on our primary analysis at 12-month follow-up using an appropriate imputation method.

#### Study 2A: longitudinal follow-up

Further statistical analyses will be conducted including the 24-month follow-up data. Three proposed analytical approaches are described below. These will support mediation analyses relevant for Study 2B.

1. Additional analysis of effectiveness: The primary analysis of intervention effectiveness will be based on the initial 12-month cluster randomised trial. The inclusion of 24-month follow-up data and the Year 2 implementation of ESAS in delayed start schools enables an additional set of analyses based on treating the study design as a two-step stepped wedge. These analyses will have marginally more power than the primary trial analyses but have increased risk of bias and are hence useful as sensitivity analyses. Under varying assumptions relating to secular trends and duration of intervention effects, models applied to data from all three time points will be used to estimate intervention effectiveness, using before and after data from both immediate and delayed start schools in a stepped wedge analysis.^54^
2. Analysis of embedded cohort: The repeat cross sections at baseline and 24 months will be among the same cohorts (S2 and S4 at baseline; S4 and S6 at 24-month follow-up). To enable within-student longitudinal analysis, students will be asked to self-generate an ID code based on stable personal characteristics; this will generate an embedded cohort with linked individual-level data, while allowing students to remain anonymous.^55^ These analyses will assess whether 24-month outcomes are related to dose or duration of intervention exposure and to identify whether 24-month outcomes vary across subgroups defined by baseline characteristics (eg, exposure to sexual harassment).
3. Within immediate start schools, change in primary and secondary outcomes over the period of 12–24 months will be calculated for each of the immediate start schools to assess whether 12-month outcomes have attenuated, sustained or amplified and whether this varies significantly across schools. Exploratory analyses guided by emerging findings from the process evaluation will assess how this variation may relate to implementation (eg, fidelity, reach and dose, acceptability and actor response), as well as identified barriers and facilitators.

#### Study 2B: process evaluation

Informed by frameworks for process evaluation grounded in a complex systems perspective,^35 36 38 39^ the evaluation will be dynamic, with analytical insights generated through earlier evaluation activities used to inform and define evaluation questions for focus at later stages. Analysis will seek to generate insights into mechanisms of action—including what works, for whom and in what circumstances^56 57^—in order to interrogate and refine the theoretical basis for ESAS. Qualitative data will be managed using NVivo software and analysed thematically, combining inductive (ie, open, in vivo) and deductive coding (ie, prespecified codes based on RQs and intervention theory). A priori codes will reflect the intervention components (eg, school self-assessment, staff training, action group), aspects of implementation (context, fidelity, dose/reach, acceptability/actor response) and anticipated mechanisms (beneficial and harmful). Codes will be developed iteratively as analysis progresses.

Drawing on a complex systems perspective, an overarching analytic goal for the process evaluation will be to synthesise evidence to build a ‘system change narrative’,^36^ explaining if and how the ESAS intervention disrupts school systems over time.^52^ Qualitative data from observations, discussions and workshops with staff and students will be used to describe and visualise (eg, using mapping software such as Kumu) indications of change within school systems (eg, to norms, relationships, processes) to refine hypotheses regarding longer term systems change and contextualise results from Study 2A. Analysis will seek to describe how school systems and the ESAS intervention adapt and coevolve over time; identify factors that either amplify or dampen change, and unanticipated consequences. We will look for a range of different examples within the data of meaningful progress and actions, including local adaptations which indicate that schools have taken ownership of GBV prevention and response and are applying the principles of ESAS in context-specific ways, while remaining consistent with the intervention theory. Ultimately, analytical insights from the process evaluation will be used to refine and visualise the intervention theory.

#### Study 3: economic evaluation

Given the known societal costs, health harms and quality of life impacts associated with GBV, an economic evaluation is integral to this research. However, currently, there are no economic evaluations of GBV interventions conducted in schools.

The economic evaluation conducted alongside the ESAS trial investigates whether the ESAS intervention is cost-effective compared with the control. It will follow standard guidance for complex population health economic evaluation,^58^,^60^ taking the complexity of the ESAS intervention into account in the design and conduct stage, using multiple economic evaluation frameworks and multiple perspectives (National Health Service and personal social services, educational and societal perspectives).

The economic evaluation alongside the ESAS RCT (Study 1) will include a within-trial analysis, exploring the cost-effectiveness of the ESAS intervention versus the comparator using a 12-month time horizon, and, pending findings from Study 1 and Study 2, a long-term model projecting likely changes in GBV episodes into long-term intersectoral costs and outcomes. An additional analysis will also use the data collected at 24 months, in line with the planned statistical analysis.

The within-trial analysis will assess cost-effectiveness of ESAS versus control using multiple evaluation frameworks:

1. A cost-utility analysis evaluating the cost-effectiveness of ESAS compared with the control in terms of additional cost per quality-adjusted life years (QALY), estimated using CHU-9D^44^; a validated age-appropriate measure validated for use with adolescents.
2. A cost-effectiveness analysis (CEA) calculating the incremental cost per reduction in sexual harassment (primary outcome^40^).
3. A cost-consequence analysis, collating costs and outcomes (within-trial QALYs, sexual harassment, coercion and violence; mental well-being, etc) in a summary table, as recommended by the National Institute for Health and Care Excellence (NICE) public health economic evaluation guidance^58^ and recent methods publications.^35 60^

The baseline and all follow-up surveys will include the preference-based quality of life instrument, the CHU-9D. They will also include a bespoke resource use questionnaire designed to measure cross-sectoral health and social care use (eg, GP and educational psychologist), community services (eg, judicial contacts) and personal health-related activities and costs (eg, private counsellors). Resources required to deliver ESAS (including displaced school activities and their associated opportunity cost) will be measured via resource use logs completed by researchers and informed by school leads. All resources measured will be valued using readily available unit costs.^61^

In line with the SAP, we will follow the approach described by Hooper *et al*,^62^ adapted to account for non-normality of health economic outcomes and costs.

We will estimate the incremental difference in total cost and outcomes using a mixed-effects generalised linear model, adjusted for school-level baseline characteristics such as school size, percentage FSM and MVP status (ie, school partaking in MVP activities). Total cost and outcomes will also be adjusted by baseline costs and outcomes to adjust for any imbalance between treatment arms.^63^ Time and treatment arm will be included as fixed effect, while schools will be included as random effect to account for clustering. Mean cost and outcomes by treatment arm, as well as incremental cost and outcomes and incremental cost-effectiveness ratio (ICER), will be predicted from the chosen econometric model and reported alongside bootstrapped SEs. The uncertainty surrounding the estimate of incremental costs, QALYs and ICERs will be investigated by use of a non-parametric bootstrap of the cost and effect pairs for 1000 iterations. This uncertainty will then be presented on the cost-effectiveness plane with a 95% CI of the bootstrapped ICER estimated. Results will be summarised using a cost-effectiveness acceptability curve to reflect the probability of ESAS being cost-effective at various willingness-to-pay (WTP) thresholds. The £20 000–£30 000/QALY threshold that NICE typically considers cost-effective will be incorporated.

Within the CEA framework, there is no accepted threshold value for changes in the primary outcome. Therefore, sensitivity analysis using different WTP values will be performed. In addition to probabilistic sensitivity analysis, several scenarios will be explored in the deterministic sensitivity analysis.

In line with the SAP, we will explore three approaches for the analysis of the 24-month follow-up data:

1. Stepped wedge analysis: we will follow best practices and recommendations described by Lung *et al*^64^ (eg, accounting for clustering, correlation between costs and outcomes, time adjustment) to conduct an analysis of costs and outcomes alongside the ESAS two-step stepped wedge design.
2. Analysis of embedded cohort: within the longitudinal study, we will conduct a secondary analysis to explore determinants of QALYs, outcomes and costs, calculated over 24 months.
3. Within intervention (immediate start) school analysis: we will assess whether outcomes and costs have sustained over time.

To support interpretation of the health economics data and acknowledging that key economic impacts are likely to be long term, we will also build an economic conceptual model^65^ with the aim of depicting the complex causal linkage between the intervention and its associated intersectoral societal costs and outcomes over the short and long terms.

Depending on the trial results, this conceptual model may inform a long-term economic model, extrapolating the 12-month effectiveness of the ESAS intervention over the long term and considering the wide spectrum of cross-sectoral impacts and costs to society of the ESAS intervention over the lifetime^66^ (eg, risk behaviours such as substance use; long-term emotional, behavioural and mental health; academic performance, employability, and teenage delinquency and crime).

The long-term model will use 24-month outcome data to predict the likely long-term cost-effectiveness of the intervention effect beyond 12 months and will be further supported by evidence from a systematic literature review.

## Ethics and dissemination

### Data management

The research team will preserve the confidentiality of participants in accordance with the 1998 Data Protection Act and 2018 General Data Protection Regulation. Data will be stored on secure information technology systems, which are backed up daily, and data access will be restricted to the trial research team. Paper documentation containing personal data will be stored in locked filing cabinets with restricted access and separate from any research data. Additionally, paper documentation will be scanned and stored in a digitised format in a restricted access drive separate from any research data. Research data and consent forms will be kept for a minimum of 10 years or for as long the research data are archived, in line with the University of Glasgow policy. Audio recordings will be transcribed in full by University of Glasgow-approved contractors with secure and confidential data transfer. Transcripts will be anonymised and stored in secure files, accessible only to named members of the research team, in a separate drive from any personal participant data. Personal contact data will be kept until the completion of the study.

### Project oversight

An independent and funder-approved TSC and DMEC will meet at least annually. The TSC will monitor trial progress, adherence to protocol, participant safety and ethical conduct. The DMEC will monitor data quality and completeness, recruitment and loss to follow-up, and advise on data analysis plans. Both the TSC and DMEC will advise on study conduct and protocol modifications. Approval for protocol amendments will be sought from the chairs of both committees. Membership will include experts in school-based interventions and trials, GBV, as well as an education professional.

### Public and patient involvement

We will establish a Young People’s Advisory Group comprising current and recent senior secondary school students with an interest in addressing school-based GBV. The group will meet online and in person to advise on study materials, processes, outputs and dissemination plans. Members will be recruited via schools undertaking ESAS and existing RCS networks. This group will meet approximately three times a year.

We will also establish an adult public involvement group, comprising school staff and parents/carers (from non-evaluation schools) to advise on study materials, outputs and dissemination plans. This group will meet online approximately two times a year.

### Ethical issues

The study has been approved by the University of Glasgow College of Medical, Veterinary and Life Sciences Ethics Committee (06/06/2023 ref 200220268).

The school leadership team consents to participate in the trial on behalf of the school, including consent to be randomised. Parents/carers will be given opportunity to opt out their child from evaluation activities, after reading an information leaflet. In all data collection activities, it will be made clear to participants that they can decline to participate, or skip specific questions, or withdraw at any time, with no negative repercussions. Informed consent for observation of intervention activities is not practical, but the presence of the observer will be explained where possible, and reassurance given regarding the anonymity of data generated by observations. In group discussions, participants will be reminded that confidentiality cannot be guaranteed and they will be asked to refrain from sharing personal stories or recounting events in a way that makes others identifiable.

### Child protection/safeguarding in the ESAS intervention and evaluation

Discussion of GBV may trigger difficult feelings for participants. To safeguard participant well-being, we will clearly indicate the topics that will be covered; provide lists of support services before and after data collection activities; offer to stop or pause if a participant becomes upset; and interact with students empathically and without expressing judgement. During qualitative data collection, if disclosures are made which suggest an immediate and serious risk of harm to a student or staff member, the researcher will notify the child protection lead for the school (via the study PI), seeking agreement from the participant as far as possible. Such disclosures will be handled confidentially via usual school safeguarding procedures. The baseline and follow-up surveys are anonymous but at the end, students will be able to leave their name via a separate secure online form if they wish a staff member to follow-up with them. The research team will securely pass these names to school staff after each survey and then delete them from their database. In this instance, safeguarding procedures apply, regardless of where the harm took place.

Serious adverse events (SAE) in this study could include incidents of sexual harassment and violence, and mental/physical ill health resulting from such incidents. Incidents of sexual harassment occur in schools regardless of ESAS and are unlikely to result from it. However, disclosures of already-occurring GBV may increase due to greater awareness and confidence to report (an aim of ESAS). We do not plan to specifically track SAEs in the trial because (a) these overlap with primary outcome data already collected by the trial; (b) reported incidents of sexual harassment are highly sensitive and confidential and schools may be unwilling to disclose and/or may view the request for information as burdensome; (c) it is unlikely that any SAE could ever be linked to the trial. We will use the health resource use questions in the student baseline survey to track the likely prevalence of SAEs and we may pick up additional information during check-in meetings between the evaluation team and ESAS staff leads.

Where SAEs become known to the study team (either directly or indirectly), these will be reported internally and to the TSC chair (and sponsor, University of Glasgow, as appropriate) within 24 hours of the study team becoming aware of an event. The University of Glasgow Clinical Trials Unit has standard operating procedures for managing and reporting any SAEs, following guidelines for Good Clinical Practice.

## Discussion

The ESAS evaluation will provide long overdue and urgently needed effectiveness, cost-effectiveness and implementation evidence on a UK intervention using a whole-school approach to address GBV, directly before it is implemented at scale. The study will also contribute empirical evidence on the scale and nature of sexual harassment in school via publication of baseline findings. This includes understanding of intersecting inequalities in experience of GBV at school, and how victimisation/perpetration of sexual harassment links with harmful gender norms,^67^ and school-level factors.

To our knowledge, this will be the first UK school-based trial to investigate the impact of whole-school approaches on school sexual harassment and GBV. Our type 1 implementation-effectiveness study design is novel in school-based evaluations. By using evaluation data to interrogate the programme theory, the trial will advance theory on mechanisms of sustainable change in whole-school approaches to tackling GBV. This will inform future decision-making regarding the transferability of ESAS to other contexts and wider roll-out.

The embedded economic evaluation within this study will identify and measure the resources needed to deliver such a GBV prevention intervention along with any short-term health and social care resource use. This study will also assess intervention impacts on school pupil’s health-related quality of life which will be combined with costs to generate cost-effectiveness evidence.

The study will enhance understanding of how systemic factors in schools can deter or facilitate interventions to address GBV in schools. We will build on learning from participatory systems mapping of factors shaping school prevention and response to sexual harassment during the pretrial pilot phase.^33^ Finally, the trial will seek to advance methods of systems-informed evaluation of complex interventions. The process evaluation and study of longitudinal outcomes are designed to explore if and how ESAS combines with the context; alters relationships between actors; facilitates cultural change; leads to shifts in the distribution of resources; and replaces other activities during and beyond the intervention period.^52^

### Trial status

Schools are being recruited from September 2023 to December 2024 and baseline student surveys will be completed in October 2023 to December 2024. Schools are being randomised on completion of their baseline student survey.

### Data availability

We are requesting participant consent to archive anonymised data in the UK data archive or University of Glasgow Enlighten Research Data repository. The data will be archived and available for sharing but will be embargoed until 2 years after the study is completed or the publication of the main study papers (whichever happens later).

### Trial documentation

The data will be held securely for a period of 10 years after the completion of the project, or longer if specified by the research funder or sponsor.

## supplementary material

10.1136/bmjopen-2024-096596 online supplemental file 1
