# Supplementary material for: Evaluating a whole-school approach to addressing gender-based violence in Scottish secondary schools (Equally Safe at School): a study protocol for a type I hybrid effectiveness-implementation trial
Source: BMJ Open. 2025 Feb 16;15(2):e096596. doi: 10.1136/bmjopen-2024-096596 (PMC11831264; doi:10.1136/bmjopen-2024-096596)
Supplement: online supplemental file 1 [file bmjopen-15-2-s001.docx]

**Supplementary File 1: Statistical power under base case assumptions and note on calculation of ICC**

**Table 1. Statistical power under base case assumptions and alternative scenarios for key sample size parameters.**

| **Scenario** | **Power** | **Effect size** | **Odds ratio** | **ICC** | **N schools** | **N pupils** |
| --- | --- | --- | --- | --- | --- | --- |
| **Base case*** | **84%** | **35% vs 27%** | **0.77** | **0.025** | **36** | **11,772** |
| Baseline-follow up primary outcome correlation (0.40) | 90% | 35% vs 27% | 0.77 | 0.025 | 36 | 11,772 |
| Loss of 10% schools (2 per arm) | 80% | 35% vs 27% | 0.77 | 0.025 | 32 | 10.464 |
| Decreased prevalence of primary outcome (32%) | 80% | 32% vs 24.6% | 0.77 | 0.025 | 36 | 11,772 |
| Increased ICC (0.05) | 82% | 35% to 24.5% | 0.70 | 0.05 | 36 | 11,772 |

***Note on calculation of ICC:*** In our pilot study (618 students from 3 schools), the ICC for our 5-item primary outcome measure of sexual harassment victimisation was 0.007, similar to those reported in other trials of adolescent GBV (0.006(1) and 0.005 (2)). Baseline data from a school-based cluster RCT of *Dating Matters*, a teen dating violence intervention, indicated that ICCs for their six teen dating violence outcomes ranged from ~.000 to .073 (3); so to prevent this trial being underpowered, we based our sample size on an ICC of 0.025 with power of 84-90%.

**References**

1. Mennicke A, Bush HM, Brancato CJ, Coker AL. Bystander Intervention Efficacy to Reduce Teen Dating Violence Among High School Youth Who Did and Did Not Witness Parental Partner Violence: A Path Analysis of A Cluster RCT. J Fam Violence. 2021 Oct;36(7):755–71.

2. Coker AL, Bush HM, Cook-Craig PG, DeGue SA, Clear ER, Brancato CJ, et al. RCT Testing Bystander Effectiveness to Reduce Violence. Am J Prev Med. 2017 May;52(5):566–78.

3. Niolon PH, Vivolo-Kantor AM, Latzman NE, Valle LA, Kuoh H, Burton T, et al. Prevalence of Teen Dating Violence and Co-occurring Risk Factors Among Middle School Youth in High-Risk Urban Communities. J Adolesc Health. 2015 Feb;56(2):S5–13.
