# Supplementary material for: Evaluating a whole-school approach to addressing gender-based violence in Scottish secondary schools (Equally Safe at School): a study protocol for a type I hybrid effectiveness-implementation trial
Source: BMJ Open. 2025 Feb 16;15(2):e096596. doi: 10.1136/bmjopen-2024-096596 (PMC11831264; doi:10.1136/bmjopen-2024-096596)
Supplement: Supplementary file 2 [file bmjopen-2024-096596.reviewer_comments.pdf]

PEER REVIEW HISTORY

BMJ Open publishes all reviews undertaken for accepted manuscripts. Reviewers are asked to complete a checklist review form and are provided with free text boxes to elaborate on their assessment. These free text comments are reproduced below.

ARTICLE DETAILS

Title (Provisional)

Study protocol for a type I hybrid effectiveness-implementation trial to evaluate a whole-school approach to addressing Gender-Based violence in Scottish secondary schools (Equally Safe at School)

Authors

Hamilton, Claire; Lewis, Ruth; Blake, Carolyn; Purvis, Anthony; Vaczy, Caroline; Deidda, Manuela; Kerr, Niamh; Waiting, Lisa; Dawson, Kathryn; Willis, Malachi; McIntosh, Emma; Taylor, Rod; Moore, Laurence; Mitchell, Kirstin

---

This article was not externally reviewed at BMJ Open. Protocols that have been independently assessed prior to submission to BMJ Open are usually fast-tracked to publication on the grounds that further substantial changes will not be possible. This independent assessment will usually be external, independent review for both a substantial grant award from a non-commercial or government funder and ethics approval.
